# A Spatial Group-Based Multi-User Full-Duplex OFDMA MAC Protocol for the Next-Generation WLAN

**DOI:** 10.3390/s20143826

**Published:** 2020-07-09

**Authors:** Meiping Peng, Bo Li, Zhongjiang Yan, Mao Yang

**Affiliations:** School of Electronics and Information, Northwestern Polytechnical University, Xi’an 710072, China; meiping@mail.nwpu.edu.cn (M.P.); libo.npu@nwpu.edu.cn (B.L.); yangmao@nwpu.edu.cn (M.Y.)

**Keywords:** WLAN, MAC, full duplex, spatial group, OFDMA, IEEE 802.11be

## Abstract

The Wireless Local Area Network (WLAN) has become a dominant piece of technology to carry wireless traffic for Internet of Things (IoT). The next-generation high-density WLAN scenario is very suitable for the development trend of the industrial wireless sensor network. However, in the high-density deployed WLAN scenarios, the access efficiency is low due to severe collisions, and the interference is diffused due to the scattered locations of the parallel access stations (STAs), which results in low area throughput, i.e., low spatial reuse gain. A spatial group-based multi-user full-duplex orthogonal frequency division multiple access (OFDMA) (GFDO) multiple access control (MAC) protocol is proposed. Firstly, the STAs in the network are divided into several spatial groups according to the neighbor channel sensing ability. Secondly, a two-level buffer state report (BSR) information collection mechanism based on *P*-probability is designed. Initially, intra-group STAs report their BSR information to the group header using low transmission power. After that, group headers report both their BSR information collected from their members and inter-group interference information to the access point (AP). Finally, AP schedules two spatial groups without mutual interference to carry on multi-user full duplex transmission on the subchannels in cascading mode. The closed-form formulas are theoretically derived, including the number of uplink STAs successfully collected by AP, the network throughput and area throughput under saturated traffic. The simulation results show that the theoretical analysis coincide with the simulation results. The system throughput of the GFDO protocol is 16.8% higher than that of EnFD-OMAX protocol.

## 1. Introduction

As the main carrier of Internet of Things (IoT), wireless local area network (WLAN) is widely used due to its advantages of low cost and easy deployment [1,2] and has become a research hotspot in industry and academia. During the past decade, with the rapid development of mobile Internet, many new applications and requirements have emerged and people’s demand for wireless traffic has increased rapidly at a compound annual growth rate of 47% from 2016 to 2021 [3], where the requirements for transmission delay and jitter are more stringent.

In order to achieve extremely high throughput, IEEE 802.11be [4] was established formally in May 2019 by the international organization of Institute of Electric and Electronics Engineering (IEEE), with the objectives to better support virtual reality, augmented reality, 4 K/8 K ultra-high definition video, remote office, and cloud computing application scenarios [5]. IEEE 802.11be continues to optimize orthogonal frequency division multiple access (OFDMA) technology and multi-user multiple input multiple output (MU-MIMO) technology introduced by IEEE 802.11ax, and takes multiple access points (multi-AP) cooperative and multi-band operation (MB-Opr) as its main technologies [6]. OFDMA technology divides channel resources into several subcarriers, and multiple successive subcarriers form a resource unit (RU). OMAX [7] is the first to propose a trigger free uplink multi-user random-access multiple access control (MAC) protocol based on OFDMA, so as to realize multi-user parallel data transmission.

However, there are still many technical bottlenecks to be solved when WLAN technology is directly applied to IoT scenarios, due to wireless scalability problems. Firstly, the network density scalability problem will appear if more and more sensors are deployed within a given network area. In other words, the low access efficiency problem of the high-density network will appear due to collisions between a large number of sensors/network devices, even if the multi-user MAC (MU-MAC) protocol, e.g., OFDMA, is employed. Secondly, the network deployment area scalability problem will appear if the sensors are deployed in a multi-hop mode over a large network area. In other words, the interference diffusion problem of the MU-MAC protocol in a high-density network will appear, where many stations (STAs)/sensors located at different positions will simultaneously transmit to AP/sink. It is harmful to exploit the spatial reuse gain. These two observations motivate us to solve the low access efficiency problem and the interference diffusion problem in high-density deployed WLAN, by exploiting the spatial grouping technology, the power control technology and full-duplex technology.

Based on the spatial grouping technology, Ref. [8] divides several spatial groups according to the spatial distance, reduces the interference diffusion problem of multi-user transmission, and improves the network area throughput. The employed spatial grouping technology in [8] can be combined with the power control technology, such that multiple spatial groups can be simultaneously transmitting or receiving, i.e., improve the spatial reuse gain. The power control technology can reduce the signal coverage, and thus eliminates the overlapping area and reduces the collisions [9,10,11,12,13]. In [14], it is proved that power control can effectively reduce the adjacent interference and improve the saturated throughput. Furthermore, if the adjacent interference is controlled and reduced by employing the power control technology, the Co-frequency Co-time Full Duplex (CCFD) technology can be exploited to enhance the overall network performance. Full duplex technology can transmit at the same time at the same frequency by using self-interference cancellation (SIC) technology [15,16], and thus the spectrum efficiency can be doubled. Therefore, it is regarded as the key piece of technology of the next generation of wireless communication [17,18].

The main contributions of this paper are summarized as follows:

1. A spatial Group-based multi-user Full Duplex OFDMA (GFDO) MAC protocol is proposed in this paper. To the best knowledge of the authors, GFDO is the first to jointly solve both the low-access efficiency problem and the interference diffusion problem in high-density deployed WLAN. This work is helpful to solve the wireless scalability problem in industrial wireless sensor networks.
  GFDO protocol is triggered by AP to collect uplink buffer state report (BSR) information and adopt a two-level BSR information collection mechanism to improve BSR collection efficiency. Meanwhile, in the second-level BSR collection process, the dynamic adjustment of the interference information between group headers is performed by detecting the power intensity, which improves the probability of forming full duplex link transmission successfully. Finally, AP schedules multi-user full duplex data transmission within each space group in a cascade manner according to the BSR information and group head interference information collected, the system throughput is greatly improved.
2. Through theoretical analysis of the proposed protocol, the closed expressions of the average nodes number of access channel, system saturation throughput and area throughput are derived.
3. The performance of the proposed GFDO protocol is compared with FD-OMAX protocol, EnFD-OMAX protocol, Mu-FuPlex protocol and OMAX protocol. The simulation results show that the theoretical analysis coincide with the simulation results, and the MAC efficiency of the proposed GFDO protocol is 16.8% higher than that of the EnFD-OMAX protocol.

The rest of this paper is organized as follows. In Section 2, the related works on full duplex MAC protocol are surveyed and the existing problems are analyzed. In Section 3, we describe the system model of GFDO protocol, including the target scenario, channel model, etc. In Section 4, we describe the process of GFDO protocol in details. In Section 5, the performance of the proposed GFDO protocol model is theoretically analyzed. Section 6 evaluates the performance of the proposed GFDO protocol, by comparing with other existing works through simulation results. This paper is summarized in Section 7.

## 2. Related Work and Motivation

According to the assumption of the network device full duplex capability, the existing MAC protocols can be divided into symmetrical and asymmetric MAC protocols. In the symmetric MAC protocols, all of the STAs have the full duplex capability. While in the asymmetric MAC protocols, only AP is assumed having full duplex capability. Therefore, we survey the existing related works according to the above two categories.

Symmetric full duplex MAC protocols usually assume that both STAs and AP have full duplex transmission capabilities. In other words, there can exists uplink and downlink transmissions simultaneously between one STA and one AP. In [19], a full duplex MAC protocol using frequency domain coordination was proposed for the next-generation WLAN. In the protocol, the AP specifies STAs to report channel information on the specified subchannel, and schedules full duplex link transmission, and STAs must have full duplex capability. In [20], a Carrier Sense Multiple Access/Collision Detect (CSMA/CD) like based multi-user full duplex MAC protocol with random competition in subchannels is proposed by using MAC frame preamble detection technology, which requires all nodes to have full duplex capability. That is, any node with data transmission request in the network sends data to the destination node as long as there are available subchannels, and the destination node transmits data to the source node at the same time.

Asymmetric full duplex MAC protocol means that AP has full duplex capability, but STAs are half duplex devices. Then, there could simultaneously exist one uplink transmission from one STA to AP, say STA A, and one downlink transmission from AP to another STA, say STA B. The communication links between STA A, AP and STA B are named as an asymmetric full duplex transmission links. With the next generation of WLAN devices miniaturization and low complexity, asymmetric full duplex MAC protocol will become a hotspot for the next-generation WLAN MAC protocol research. Q. Qu [21] et al. believed that in the next-generation WLAN full-duplex scenario, only the AP should have full duplex capability and STAS could not. Then, based on this assumption, the Mu-FuPlex [22] protocol and the PCMu-FuPlex [23] protocol based on AP pure scheduling are presented. However, the multi-user MAC protocol based on pure scheduling leads to high overhead in the process of BSR information collection. Therefore, the authors’ previous studies have proposed a multi-user full duplex MAC protocol based on multi-user random access for the next-generation high-density deployment scenarios in FD-OMAX protocol [24], EnFD-OMAX protocol [25], which greatly improves the system throughput.

In summary, full duplex MAC protocol can increase system throughput, and Table 1 compares these MAC protocols from the following aspects, in terms of topology, contention based, performance metric, key features and evaluation method. However, all of the existing works do not take into account the collection efficiency of the uplink transmission requirements, and the STAs’ location dispersion of multi-user transmission leads to serious interference diffusion. On the one hand, if the collection efficiency of uplink transmission requirements is low, the probability of forming a full duplex transmission pair is low, which directly leads to the low efficiency of full duplex MAC protocol. On the other hand, if the STAs positions involved in full duplex transmission are scattered, the overall interference area expands, which directly affects the area throughput of the system. Therefore, the access efficiency and interference diffusion are significant problems to be addressed, in the design of full duplex MAC protocol for the next-generation high-density WLAN.

Based on our overview of the MAC protocol for CCFD transmission, this paper assumes that STAs can detect the received power intensity on the subchannel. Combining power control and spatial grouping technology, a spatial group-based multi-user full duplex OFDMA MAC protocol is proposed. Firstly, the AP divides the STAs into several spatial groups according to the neighbor channel sensing capability (NCSC) [26]. In spatial group, the STAs use the power control technology to report the BSR information to the group header in the low power mode. Secondly, group headers report the BSR information collected in this round and its own BSR information to the AP. In this process, the interference information among the group headers is dynamically updated. Finally, AP schedules multi-user full duplex transmission in the spatial group according to the interference information between spatial groups. The GFDO protocol uses trigger frames to manage the uplink/downlink transmission, which is compatible with the draft standard of IEEE-802.11ax/be.

## 3. System Model

This section mainly introduces the system model of GFDO protocol proposed in this paper. Before that, in order to better describe the system model, we firstly present the following definitions.

**Definition 1**: *Space Group (SG) is composed of STAs with the same NCSC. In a high-density deployed scenario, there are multiple SGs in a single basic service set (BSS) designated by AP.*

**Definition 2**: *Group Header (GH), designated by AP, is responsible for collecting BSR information of group members (GM) in a SG and interference information between GHs, and then reporting BSR information and interference information to AP.*

**Definition 3**: *Group Members (GM) are designated by AP according to the NCSC. The STAs with the same NCSC form a SG.*

**Figure 1:**
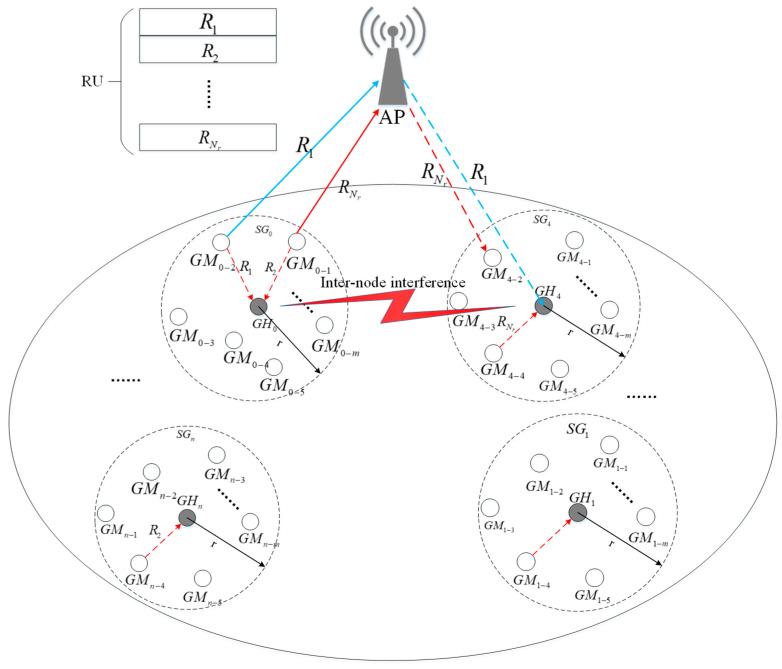
Full duplex communication network scenario under high-density deployment.

**Definition 4**: *Group Full Duplex Transmission (GFDT): AP schedules GM in the uplink SG and in the downlink SG to form a GFDT according to the interference information between the GHs reported by GHs. As shown in Figure 1,* $GH_{0}$ *, AP and* $GH_{4}$ *form a GFDT, i.e., a spatial group-based asymmetric full duplex transmission link.*

In the GFDO protocol, the single BSS scenario for the next-generation WLAN is considered, which is composed of an AP with full duplex capability and several STAs with half duplex capability. As shown in Figure 1, AP is deployed at the geometric center of BSS, GMs are randomly distributed in several spatial groups under the coverage of AP, and the set of STAs is denoted as $S=\left\{S_{1},S_{2}\cdots S_{N_{s}}\right\}$. $N_{s}$ GMs are divided into $G_{s}$ SGs, denoted as $G=\left\{G_{1},G_{2}\cdots G_{G_{s}}\right\}$. Each SG contains one GH and $N_{g}$ GMs. Suppose the whole bandwidth of the WLAN system is divided into $N_{r}$ resource unit (RU), which is denoted as $R=\left\{R_{1},R_{2}\cdots R_{N_{r}}\right\}$.

The multi-user full duplex MAC protocol designed in this paper belongs to the asymmetric full duplex MAC protocol, as shown in Figure 1. $GM_{0-1}$ and $GM_{0–2}$ in $SG_{0}$ have uplink transmission requirements, $GM_{4–2}$ and $GH_{4}$ in $SG_{4}$ have downlink transmission requirements. AP allocates channel resources $R_{1}$ to $GM_{0–1}$ and $GM_{4–2}$, and channel resources $R_{N_{r}}$ to $GM_{0–2}$ and $GH_{4}$ to form full duplex transmission. The uplink transmission requirements of GMs in a SG are collected by GH and reported to the AP. In our model, in order to maximize the system throughput, we need to achieve the following conditions: (1) the collection efficiency of uplink transmission demand; (2) the collection of interference intensity between SGs.

## 4. Protocol Description

### 4.1. The Basic Idea of GFDO Protocol

**Figure 2:**
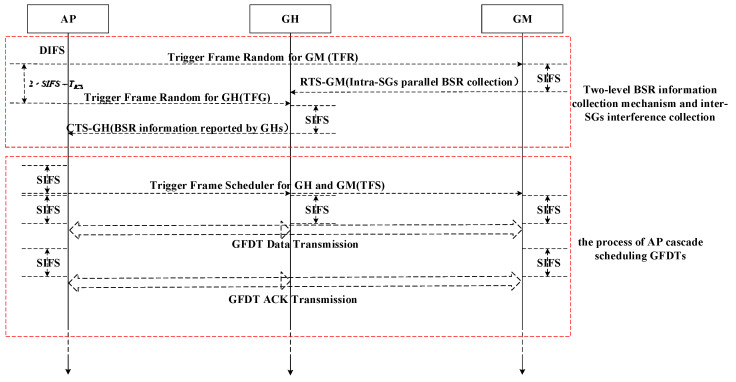
Overall flow chart of group-based multi-user full duplex orthogonal frequency division multiple access (OFDMA) (GFDO) protocol.

The GFDO protocol is an asymmetric full duplex MAC protocol for STAs random-access triggered by AP. The overall protocol flow is shown in Figure 2, including two stages, i.e., the two-level BSR information collection mechanism and inter-SGs interference collection, and the process of AP cascade scheduling GFDTs. Among them, the two-level BSR information collection mechanism is adopted for the uplink transmission demand collection. It includes the BSR information collection of GMs in SG and the BSR information collection of SG.

- In the first level, GH can be seen as a virtual gateway, which is used to collect the BSR information of GMs in SG and record the interference information of other GH. Because the formation of SG in GFDO protocol is based on the NCSC, the GMs in each SG can independently and synchronously report BSR information to the GH. Therefore, GFDO protocol can effectively improve the efficiency of BSR information collection, so as to improve the system throughput.
- In the second level, GHs report their GMs and their own BSR to AP if they have collected BSR in the first level. Otherwise, the GHs, with no collected BSR, dynamically update the inter-node interference intensity, which is almost real-time and improves the formation probability of a full duplex link.

It is worth noting that the compatibility of GFDO protocol with IEEE 802.11be is fully considered in the design, which has better backward compatibility.

The key technologies of GFDO protocol are spatial grouping process and parallel collection of STAs uplink requirements in SG. First, we adopt the NCSC [26] to form the spatial groups. With NCSC, it means that if the distance between two nodes, say A and B, not exceed r (r refers to cell radius), then they will sense the channel activity status with the same probability. We define the receiving power of node A and node B at time t as $P_{A}\left(t\right)$ and $P_{B}\left(t\right)$, respectively, assuming that $P_{d}\left(t\right)$ is a difference of received power between receiving node A and receiving node B at time t. Then, the neighbor channel sensing ability can be expressed by Formula (1), where $\sigma_{d}{}^{2}$ is the variance of $P_{d}\left(t\right)$, $\sigma_{A}{}^{2}$ is the variance of $P_{A}\left(t\right)$ and $\sigma_{B}{}^{2}$ is the variance of $P_{B}\left(t\right)$. When ξ tends to 1, the nodes can accurately sense each other’s channel state. That is, as long as the distance between nodes is close enough, their channel sensing ability is consistent. Through theoretical analysis and simulation verification, [26] proved the existence of neighbor channel awareness in high-density deployment scenarios and proved the correlation of the channel awareness ability of neighbor nodes. When the AP divides space groups, it refers to the analysis results of [26] and forms an SG with the same or similar NCSC. The implementation process is feasible.

(1) $$\xi =1-\frac{\sigma_{d}{}^{2}}{\sigma_{A}{}^{2}+\sigma_{B}{}^{2}}$$

As the core technology of IEEE 802.11 standard, power control has been put forward in the early standard version, e.g., IEEE 802.11 g. By adjusting the transmission power of AP and STAs, the collision and energy consumption can be reduced. The transmission power is reduced and the concurrent transmission rate in the network is increased [27]. Therefore, the spatial group BSR information collection proposed in this paper can achieve independent and parallel BSR collection process in each spatial group in the network by adjusting the transmission power of appropriate intra group BSR collection due to the same neighbor channel sensing capability of STAs in the group.

### 4.2. Protocol Process Description

The specific process of GFDO protocol is as follows.

- After AP successfully access into the channel, it starts the first level BSR information collection, i.e., GMs BSR information collection, by sending the BSR poll-trigger (BSRP-TFR) frame to start GMs uplink transmission demand collection in SG. Once the GMs receive BSRP-TFR frame, they report BSR to their respective GHs, with an power control based P-probability OFDMA random access method. In other words, once a GM receives BSRP-TFR, it randomly selects one RU and report its own BSR to its GH with the probability of P, and with a reduced transmission power. The reduced transmission power can only guarantee the BSR transmission to its own GHs, but with no harmful interference to other GHs.
- After the first-level BSR information collection, i.e., GMs BSR information collection, AP sends the BSRP-TFG frame to start the second-level BSR information collection. Once GHs receive the BSRP-TFG frame, they also adopt the P-probability OFDMA random access method to report two kinds of information to the AP. The first one is the BSR information collected in the first-level BSR information collection from the GMs, and its own BSR information if any. The second one is the interference information between SGs. However, if the GHs do not have any collected BSR information in the first-level BSR information collection, they will be in an idle state and do not report to AP. The idle GHs detect and record the interference intensity of other GHs on the subchannel.
- After two-level BSR information collection completion, the cascaded spatial group full duplex transmission is started. AP allocates RU resources according to the collected BSR and interference information among the GHs, and schedule a GFDT to start the multi-user full duplex transmission in cascaded mode.

#### 4.2.1. Two-Level BSR Information Collection Mechanism

In the GFDO protocol, a two-level reporting mechanism is adopted for uplink BSR collection. The first-level BSR collection is that GHs as a virtual gateway independently and synchronously receive the BSR information reported by GMs in SG. The second-level AP collects the BSR information of GHs and the BSR information of GMs collected by GHs.

In the first level, the AP sends the BSRP-TFR frame to trigger all GMs to access the channel by OFDMA multiple access mode based on the joint control strategy of *P*-probability and power control. GHs are not allowed to compete with the channel and is in the receiving state. GMs use the same channel resource to report the BSR information to the corresponding GH independently and synchronously, so as to reduce the probability of access collision in the high-density deployment scenario. In addition, GMs randomly select an RU to send a request to send (RTS) frame after obtaining the transmission opportunity, which it does not send the RTS frame on the whole channel, thus further improving the probability of successfully accessing channel. As shown in Figure 1, all GMs in $G_{0},\cdots ,G_{n}$ are in the listening channel state. Once the BSRP-TFR frame sent by AP is listened to, after an short inter frame space (SIFS) time, all GMs randomly select a RU in range of $\left[0,RU_{N_{r}}\right)$, and send the RTS frame to the respective GH based on *P*-probability on the selected RU. All of the GHs successfully receive the BSR frame sent by STAs in its SG and record the BSR information received. The BSR information set is recorded as follows:

$$GH_{bsr}=\left\{BSR_{N_{1}},BSR_{N_{2}}\cdots BSR_{N_{g}}\right\}$$

AP sets a timer after sending the BSRP-TFR frame, the length of which is the time of the first-level BSR information collection, defined as $\tau =t_{sifs}+t_{rts}+\Delta$, where $t_{sifs}$ is SIFs time, $t_{rts}$ is RTS frame transmission time, and Δ is signal transmission time. After the timeout of the timer, AP sends the BSRP-TFG frame after an SIFS time to trigger the second-level BSR information collection.

During the second-level BSR information collection, GMs are not allowed to access the channel, GHs use OFDMA multiple-access mode based on *P*-probability to compete for accessing the channel. Firstly, after receiving the BSRP-TFG frame, all of the GHs randomly selects an RU resource block within the range of $\left[0,RU_{N_{r}}\right)$, and sends the group clear to send (G-CTS) frame on the selected RU. The G-CTS frame includes: BSR information collected at the first level, BSR information of the node itself and interference intensity information between SGs. As shown in Figure 1, the $GH_{0},\cdots ,GH_{n}$ report BSR information to the AP in the second-stage of BSR information collection. AP records all BSR information collected in this round, and its set is expressed as:

$$AP_{bsr}=\left\{\begin{array}{l} \{G_{0},\{(GM_{0,1},bsr),(GM_{0,2},bsr),\cdots ,(GM_{0,N_{g}},bsr)\}\},\cdots ,\\ \left\{G_{s},\{(GM_{s,1},bsr),(GM_{s,2},bsr)\cdots (GM_{s,N_{g}},bsr)\}\right\} \end{array}\right\}$$

The detailed flow of two-level BSR information collection mechanism is shown in Algorithm 1.


**Algorithm 1: Two level BSR information reporting mechanism**

1: **Global initialization: //**According to Equation (1), all of nodes in the network are divided into $G_{s}$ space groups, each of which contains one GH and $N_{g}$ GMs. Set the value of *P*-probability p.
2: **Step 1:** All GMs start the first level BSR information collection after receiving the BSRP-TFR frame sent by AP.
3:  a. All GMs pick two random value to prepare access channel and Check if data queue $Q_{size}$ is not empty.
   P=Random[0,1]
   $RU_{index}=Random[0,RU_{N_{r}})$
4.   b. **IF**
P−p≥0 and $Q_{size}>0$
**THEN**
5.    GMs access channel on $RU_{index}$ to send RTS frame in low power mode.
6.    **ELSE**
7.    GMs remain idle state.
8.    **ENDIF**
9.   c. GHs receive the RTS frame sent by GMs in the group, and records the received BSR information $GH_{bsr}$.
10. **Step 2:** After receiving the BSRP-TFG frame, GHs is ready to start the second level BSR information collection
11. a. All GHs pick two random value to prepare access channel and Check if data queue $Q_{size}$ is not empty.
  P=Random(0,1]
  $RU_{index}=Random[0,RU_{N_{r}})$
12.  b. **IF** (P−p≥0 and $Q_{size}>0$) or (P−p≥0 and $GH_{bsr}>0$) **THEN**
13.   GHs access channel on $RU_{index}$ to send G-CTS frame in full power mode.
14.  **ELSE**
15.   GHs keep receiving status and monitor the power intensity on RUs
16.  **ENDIF**
17. c. The AP receives the G-CTS frame sent by GHs, and records all BSR information collected $AP_{bsr}$.

In summary, GFDO protocol adopts a two-level BSR information collection mechanism. On the one hand, AP divides nodes into several SGs according to NCSC, and specifies the transmission power of nodes in first level information collection. Therefore, when all of the GHs collect BSR information in respective SG simultaneously, there is no interference between each other, and the spatial reuse gain of the system channel resources is improved, so as to improve the area throughput of WLAN. On the other hand, OFDMA multiple-access mode based on *P*-probability strategy is adopted. Firstly, once a GM receives the trigger frame sent by AP, it randomly selects an RU within the range of the maximum resource block of the system. However, it does not immediately send the BSR information frame. The GMs that win the transmission opportunity based on *P*-probability strategy send the BSR information frame on the selected RU, so as to improve the node number of successful access channel, so as to further improve the system throughput of WLAN. Especially in the high-density network scenario for next-generation WLAN, massive amounts of nodes and APs are deployed in a limited area, the access collision probability is more serious. However, the GFDO protocol divides nodes into groups and competes for channels independently, which can significantly alleviate the access collision caused by a large number of nodes simultaneous access channels.

The collection of interference intensity between nodes is one of the problems to be solved in the design of full duplex MAC protocol. In order to improve the success probability of the full duplex transmission pair, the real-time inter-node interference intensity information as a key factor should be considered in full duplex link pairing. Therefore, the AP obtaining real-time inter-node interference intensity information is the cornerstone of improving the success probability of full duplex transmission.

In the second stage of BSR collection, GHs in an idle state monitor the channel and record the interference signal intensity of other GHs to this node. See Step 2 of Algorithm 1 for the collection and processing flow. As shown in Figure 1, assuming that $GH_{0}$ and $GH_{4}$ obtain access channel opportunities, G-CTS frames are sent in $RU_{3}$ and $RU_{1}$ respectively, while other GHs, such as $GH_{1}$ and $GH_{n}$, record the received power on different RU, and the set represents:$I_{GH}=\left\{\left(G_{0},p),(G_{1},p)\cdots (G_{s},p\right)\right\}$, where p is the received power intensity of G-CTS frame on RU. Each time the new interference signal strength of the GH is detected, the corresponding interference intensity information is dynamically updated. In the second-level BSR information collection process, the GH writes the inter-node interference information into the G-CTS frame and reports it to the AP. After the AP receives the G-CTS frame sent by GH, it maintains a GH interference intensity information table, which set is:

$$I_{AP}=\left\{\begin{array}{l} \{G_{0},\{(G_{1},p)\cdots (G_{s},p)\}\},\{G_{1},\{(G_{0},p)\cdots \\ \left(G_{s},p)\}\}\cdots \{G_{s},\{(G_{0},p)\cdots (G_{s-1},p)\}\right\} \end{array}\right\}$$

Because AP groups nodes according to NCSC, the GH and GMs in SG have the same channel sensing ability, that is, the interference intensity information of the GMs in the SG and the GMs in other SG can be represented by interference intensity information of the GH and other GHs.

#### 4.2.2. Group Full Duplex Transmission in a Cascading Method

According to the description in the previous chapter, once the collection of uplink transmission demand and interference intensity information are complete, AP starts to schedule a GFDT in a cascading method. When the AP establishes a GFDT, it uses a Formula (2) to determine whether the power interference value transmitted by the uplink SG meets the signal to interference plus noise ratio (SINR) threshold received by the downlink SG, and form the pairing of the GFDT. Otherwise, a full duplex link cannot be formed. As shown in Figure 1, the power interference value of the uplink group $G_{0}$ meets the SINR threshold received by the downlink group $G_{4}$, which can form a GFDT.

(2) $$SINR_{fd}=\frac{P_{r,DL(m)}}{N_{0}+I_{DL(m)}+\Delta P}$$

where $P_{r,DL(m)}$ is the receiving power of downlink SG receiving downlink data on $RU_{m}$ resource block. $I_{DL(m)}$ represents the interference power of uplink SG to downlink SG when it forms a full duplex transmission pair on the $RU_{m}$ resource block. ΔP is the protection power value, this is, the average difference between GHs.

After AP completes the computation of the group full duplex transmission pairing, it sends the BSRP-TFC frame scheduling a GFDT. In the scheduling process, the SG is regarded as an entity, and Algorithm 2 shows the pseudo code of the GFDT scheduling in a cascading method, where the selection of the SINR threshold is based on [22]. AP schedules the group full duplex transmission according to the BSR information collected in this round. During the scheduling process, if the uplink demand collected by a group is greater than the number of RU in the system, the AP performs the cascade scheduling in this group until all nodes with uplink demands are collected in this group. If the number of uplink requirements collected by this group is less than the total number of RUs in the system, the out of group cascade scheduling will be performed until all uplink requirements collected are transmitted and the full duplex transmission of this group is completed. The unit of data transmission is the spatial group, and the information collected by BSR is taken as the benchmark, that is, scheduling members of two spatial groups that do not interfere with each other to form asymmetric group full duplex data transmission.
**Algorithm 2: Group full duplex transmission in a cascading method****INPUT:** Uplink transmission demand set $AP_{bsr}$, Downlink transmission demand set DLset, Inter-SGs interference intensity $I_{AP}$, SINR threshold $SINR_{throld}$**OUTPUT:** Find $\left\{FD_{n},S\left(GM_{i,ul},GM_{j,dl},RU_{index}\right)\right\}$$\forall GM_{i,ul}\in G_{i},GM_{j,dl}\in G_{j},i\neq j$, and $RU_{index}\in [0,RU_{N_{r}})$, $FD_{n}$ is cascade number
1: **Initialization:**
2:  ULsize = size of $AP_{bsr}$ // Number of SGs BSR information collected
3:  DLsize = size of DLset // Number of Downlink SGs transmission demand
4:  $RU_{index}$ = 0
5:  CasNum = 1
6: **FOR** ULsize **DO**
7: **FOR** DLsize **DO**
8:   Calculate the SINR of $GH_{ULsize}$ and $GH_{DLsize}$ according to Equation (2) and Inter-SGs interference intensity $I_{AP}$
9:   **IF**
$SINR>SINR_{throld}$
**THEN**
10:    in Size = size of $AP_{bsr}(ULsize)$ // Number of BSR information in a single SG
11:    **FOR** inSize **DO**
12:     Write full duplex link sets: $\left\{CasNum,S\left(GM_{i,inSize},GM_{j,inSize},RU_{index}\right)\right\}$
13:     $RU_{index}$++
14:     **IF**
$RU_{index}\geq RU_{N_{r}}$
**THEN**
15:      CasNum++
16:      $RU_{index}$ = 0
17:     **ENDIF**
18:    **ENDFOR**
19:   **ENDIF**
20:  **ENDFOR**
21: **ENDFOR**


#### 4.2.3. Scheduling Strategy of Group Full Duplex Transmission

The GFDO protocol belongs to the AP centralized scheduling MAC protocol. According to the above sections, AP controls the BSR information and intergroup interference strength of the whole network node. In order to simplify the proposed protocol model, the fixed rate data transmission is adopted, so that the maximum full duplex transmission link pair can be formed in one transmission process is the optimization goal. Therefore, the bipartite graph matching algorithm is adopted to optimize the maximum full duplex link pair. Because only GHs participate in bipartite graph matching in GFDO protocol, and its computation complexity is $\Theta \left(n_{GH}{}^{2}\right)$, while all nodes participate in matching in other protocols, and its computation complexity is $\Theta \left[{\left(n_{GH}\cdot N_{g}+n_{GH}\right)}^{2}\right]$.

Because $n_{GH}{}^{2}$ is less than ${\left(n_{GH}\cdot N_{g}+n_{GH}\right)}^{2}$, the computation complexity of GFDO protocol is better than other protocols. Of course, the bipartite graph matching optimization algorithm is not the best method. It is our future research work to joint *P*-probability, User-AP Association power control and Modulation and Coding Schemes (MCS) to achieve the maximum throughput.

## 5. Performance Analysis

Based on the proposed GFDO protocol model, this section analyzes the average number of nodes in each round of the access channel and the system throughput under the ideal channel. During the analysis, we assume that the data queue of AP and STA is always non-empty and the network is saturated. Firstly, we define three variables used in the analysis of two-level BSR information collection: *P*-probability value p, the number of spatial groups G, and the number of nodes in the group $N_{g}$. Secondly, the two-level BSR information collection process is two independent collection processes. Therefore, we analyze them separately. Finally, the closed expression of saturated throughput and the average number of STAs access channel are obtained, which provides effective theoretical support for the GFDO protocol proposed in the paper.

### 5.1. Analysis of the Average Number GMs of Access Channels in a Single SG

The success collection of BSR on RU depends on whether there is only one GM sending BSR request on the RU and no other GM sending BSR request on the RU. Let $P_{gm}\left(i\right)$ to access channel probability for i GM in each SG:

(3) $$P_{gm}\left(i\right)=\{\begin{array}{ll} 1 & if\;\;\;\;\;p=1\\ \left(\begin{array}{c} N_{g}\\ i \end{array}\right)p^{i}{\left(1-p\right)}^{N_{g}-i} & if\;\;\;\;\;p\in \left(0,1\right) \end{array}$$

where $N_{g}$ is the number of GMs. For each SG, there are i GMs sending a BSR request access channel in the current time slot, and each GM independently selects an RU. The probability of successful access channel is for each RU if and only if only one GM sends a BSR request on the RU, as shown in Equation (3):

(4) $$P_{ru}=\frac{1}{M}{\left(1-\frac{1}{M}\right)}^{i-1}$$

The probability of GM sending BSR request on M RUs can be considered as M independent events with the same distribution, so the probability of average access channel in a competitive process is:

(5) $$P_{m\text{-}ru}=MP_{ru}$$

Therefore, it can be concluded that the average number of GM of successful access channels in a single SG is:

(6) $$N_{gm}=\sum_{i=1}^{N_{g}}iP_{m\text{-}ru}P_{gm}\left(i\right)$$

### 5.2. Analysis of the Average Number GHs of Access Channels in the System

In GFDO protocol, the way in which GMs compete for the access channel is the same as the GHs competing for the access channel. Define $P_{gh}\left(i\right)$ as the probability of preparing access channel for i GHs in the network:

(7) $$P_{gh}\left(i\right)=\{\begin{array}{ll} 1 & if\;\;\;\;\;\;\;\;\;\;p=1\\ \left(\begin{array}{c} G\\ i \end{array}\right)p^{i}{\left(1-p\right)}^{G-i} & if\;\;\;\;\;\;\;\;\;p\in \left(0,1\right) \end{array}$$

As the GHs reports BSR information, M RU OFDMA mode is also used to access the channel. Similarly, the average number of GHs successfully accessed in a competition process is:

(8) $$N_{gh}=\sum_{i=1}^{G}iP_{m\text{-}ru}P_{gh}\left(i\right)$$

According to the two-level information reporting mechanism proposed in this paper, that is, GMs successfully sends BSR request to the GH, and the GH successfully sends BSR request to AP, and the first-level transmission and the second-level transmission are independent events, the average number of BSR request successfully transmitted in the two-level information collection mechanism is:

(9) $$N_{ap}=\sum_{i=1}^{G}iP_{m\text{-}ru}P_{gh}\left(i\right)\sum_{i=1}^{N_{g}}iP_{m\text{-}ru}P_{gm}\left(i\right)+\sum_{i=1}^{G}iP_{m\text{-}ru}P_{gh}\left(i\right)$$

### 5.3. Saturated throughput Analysis

**Figure 3:**
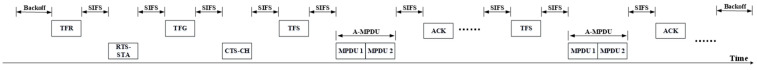
Transmission time mechanism of GFDO protocol.

According to the description in the previous chapter, the GFDO protocol can be regarded as a pure scheduling MAC protocol. In this section, based on the proposed GFDO protocol working in the ideal channel, the system throughput of the GFDO protocol is analyzed. The total length of a transmission can be divided into BSR information collection time and full duplex data transmission time, as shown in Figure 3. According to the description in Figure 3, define the time length of two stages, as shown in Equations (10) and (11).

(10) $$T_{bsr}=T_{tfr}+T_{rts}+T_{tfg}+T_{cts}+3\cdot T_{sifs}+T_{difs}$$

(11) $$T_{data}\left(i\right)=N_{gh}\cdot \left(T_{tfs}+T_{ack}\left(i\right)+T_{payload}\left(i\right)+3\cdot T_{sifs}\right)$$

where $T_{tfr}$ is the transmission time length of BSRP-TFR frame, $T_{rts}$ is the time length for STA to report BSR frame, $T_{tfg}$ is the transmission time length of BSRP-TFG frame, $T_{cts}$ is the time length of BSR frame reported by GH, $T_{tfs}$ is the transmission time length of TFS frame, $T_{ack}\left(i\right)$ and $T_{payload}\left(i\right)$, respectively, represent the length of time when there are i pairs of full duplex links transmitting on different RU at the same time in the process of full duplex data transmission. Then, the expression of closed throughput in the saturated state is:

(12) $$S=\{\begin{array}{c} \frac{E\sum_{i=1}^{G}iP_{m\text{-}ru}P_{gh}\left(i\right)\left(\sum_{i=1}^{N_{g}}iP_{m\text{-}ru}P_{gm}\left(i\right)\right)}{T_{bsr}+\sum_{i=1}^{G}P_{m\text{-}ru}P_{gh}\left(i\right)\sum_{i=1}^{N_{g}}P_{m\text{-}ru}P_{gm}\left(i\right)T_{data}\left(i\right)}\;\;\;\;\;\;\;\;\;\;\;\;\;\;\;\;\;\;\;\;\;\;\;\;\;\;\;\;\;\;\;\;\;\;\;\;\;\;\;\;\;\;\;\;\;\;\;\;\;\;\;\;\;\;\;\;\;\;\;\;\;\;\;\;\;\;\;\;\;if\;\;\;\;\;G=1\\ \frac{2E\sum_{i=1}^{G}iP_{m\text{-}ru}P_{gh}\left(i\right)\left(\sum_{i=1}^{N_{g}}iP_{m\text{-}ru}P_{gm}\left(i\right)\right)}{T_{bsr}+\sum_{i=1}^{G}P_{m\text{-}ru}P_{gh}\left(i\right)\sum_{i=1}^{N_{g}}P_{m\text{-}ru}P_{gm}\left(i\right)T_{data}\left(i\right)}\;\;\;\;\;\;\;\;\;\;\;\;\;\;\;\;\;\;\;\;\;\;\;\;\;\;\;\;\;\;\;\;\;\;\;\;\;\;\;\;\;\;\;\;\;\;\;\;\;\;\;\;\;\;\;\;\;\;\;\;\;\;\;\;\;\;\;\;\;if\;\;\;\;\;G\neq 1 \end{array}$$

### 5.4. Area Throughput Analysis

Area throughput refers to the amount of data sent by all nodes in the network in unit time and unit region, which is numerically equal to the ratio of the system throughput to the interference region of the sending node. In the paper, we assume that the interference region of the sending node is the region of the carrier sensing region when the node sending the frame, and the carrier sensing region radius is $r_{cs}$, and the NCSC radius is $r_{n\csc}$, then the interference area of the group full duplex transmission can be expressed by Equation (13).

(13) $$S_{n\csc}=\pi {\left(r_{cs}+r_{n\csc}\right)}^{2}$$

According to the throughput Equation (12), the area throughput can be represented by Equation (14).

(14) $$S_{reg}=\{\begin{array}{c} \frac{E\sum_{i=1}^{G}iP_{m\text{-}ru}P_{gh}\left(i\right)\left(\sum_{i=1}^{N_{g}}iP_{m\text{-}ru}P_{gm}\left(i\right)\right)}{\left(T_{bsr}+\sum_{i=1}^{G}P_{m\text{-}ru}P_{gh}\left(i\right)\sum_{i=1}^{N_{g}}P_{m\text{-}ru}P_{gm}\left(i\right)T_{data}\left(i\right)\right)\cdot \pi {\left(r_{cs}+r_{n\csc}\right)}^{2}}\;\;\;\;\;\;\;\;\;\;\;\;\;\;\;\;\;\;\;\;\;\;\;\;\;\;\;\;\;\;\;\;\;\;\;\;\;\;\;\;\;\;\;\;\;\;\;\;\;\;\;\;\;\;\;\;\;\;\;\;\;\;\;\;\;\;\;\;\;if\;\;\;\;\;G=1\\ \frac{2E\sum_{i=1}^{G}iP_{m\text{-}ru}P_{gh}\left(i\right)\left(\sum_{i=1}^{N_{g}}iP_{m\text{-}ru}P_{gm}\left(i\right)\right)}{\left(T_{bsr}+\sum_{i=1}^{G}P_{m\text{-}ru}P_{gh}\left(i\right)\sum_{i=1}^{N_{g}}P_{m\text{-}ru}P_{gm}\left(i\right)T_{data}\left(i\right)\right)\cdot \pi {\left(r_{cs}+r_{n\csc}\right)}^{2}}\;\;\;\;\;\;\;\;\;\;\;\;\;\;\;\;\;\;\;\;\;\;\;\;\;\;\;\;\;\;\;\;\;\;\;\;\;\;\;\;\;\;\;\;\;\;\;\;\;\;\;\;\;\;\;\;\;\;\;\;\;\;\;\;\;\;\;\;\;if\;\;\;\;\;G\neq 1 \end{array}$$

## 6. Performance Evaluation

### 6.1. Simulation Scene and Parameter Setting

In order to verify the efficiency of the GFDO protocol, a system and link level integrated simulation platform based on NS2 is built. In the simulation, we assume that in the process of receiving frames, as long as the received SINR is greater than or equal to the SINR threshold, the frame can be received successfully. In all simulations, the traffic flow is saturated, that is, each GM and GH always have uplink fame to AP and AP always has downlink frame to GM or GH. The time of each simulation is set to 50 s, and the average simulation result of 5 times of repeated simulation is finally taken. According to IEEE 802.11ax protocol draft, a 20 MHz full channel is divided into nine RUs, so, in the simulation scenario, nine STAs access channels at the same time are supported.

In the paper, we only consider the single BSS scenario. AP deployed in the geometric center of 100 × 100 m^2^ simulation area, the number of GMs in each group $N_{g}$ is 1, 3, 5, and the number of GH starts from 1, and increases gradually to 20 at one interval. The GHs were randomly distributed within the coverage of AP, and the GMs in SG were randomly distributed within the NCSC of the GH. According to the analysis of [26], the coverage of GH is set to 5 m. And the fixed QPSK modulation and 1/2 coding is used for data packet in all simulations. Other network parameter settings are shown in Table 2. The efficiency of BSR collection under different network scales, *P*-probability and number of GHs are verified. At the same time, the system performance of MU-FuPlex protocol and OMAX protocol is compared and verified, and its throughput is greatly improved. All the numerical simulation results verify the correctness of the proposed GFDO protocol.

BSR collection efficiency is the key performance metric in the GFDO protocol. BSR collection efficiency is a measure of system network capacity, which represents how many STAs participate in full duplex transmission at the same time. Therefore, the subsequent theoretical analysis and simulation verification in GFDO take BSR collection efficiency as the metric parameter to measure the system performance.

### 6.2. Simulation Result

#### 6.2.1. Analysis of the Average Number of STAs Access Channel

**Figure 4:**
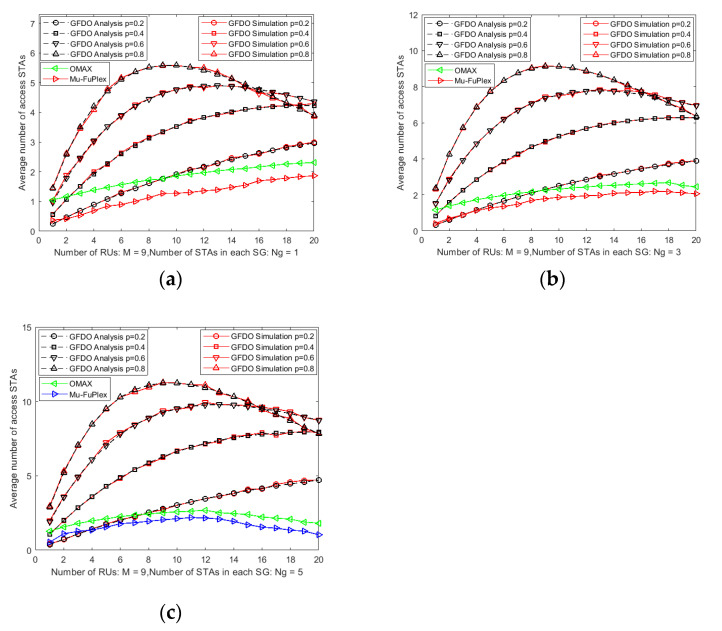
Analysis of the number of access STAs with a different *P*-probability. (**a**) The average number of access STAs vs. Number of STAs in each SG is 1; (**b**) The average number of access STAs vs. Number of STAs in each SG is 3; (**c**) The average number of access STAs vs. Number of STAs in each SG is 5.

The average number of STAs access network is an important performance metric to measure network performance. The more the average number of STAs access channel, the more uplink BSR information that AP collects, and the higher the scheduling efficiency of the GFDO protocol. The GFDO protocol model proposed combines the advantages of pure scheduling and random access, uses *P*-probabilistic in the BSR information collection stage and pure scheduling cascade transmission in the full duplex data transmission stage, which greatly improves the system performance. Figure 4 shows the average number of STAs access networks with a different *P*-probability. From Figure 4, we can see that the *P*-probability is 0.2, 0.4, 0.6, and 0.8, the number of GHs is 1–20, and the number of STAs in the SG is 1, 3, and 5 in Figure 4a–c, respectively. From the theoretical analysis and simulation curves in Figure 4a–c, the two curves are basically the same, which effectively verifies the correctness of the GFDO protocol. It provides reliable theoretical support for the proposed GFDO protocol.

As can be seen from Figure 4a, compared with the Mu-FuPlex and OMAX protocols, in the small-scale network scenario, due to the small competition conflict between the OMAX and Mu-FuPlex protocols, when the selected *P*-probability value is less than 0.8, the number of node access channels is better than the GFDO protocol. With the increase of the network scale, as shown in Figure 4a–c, the competition conflicts become more and more serious. As shown in Figure 4a–c, when *p* = 0.6 or 0.8, the number of access STAs of GFDO protocol based on the two-level BSR information collection mechanism is obviously better than that of Mu-FuPlex and OMAX protocol. The MU-FuPlex protocol adopts the competitive access mode of AP scheduling, and its average access channel number is less than that of OMAX.

#### 6.2.2. Saturated Throughput Analysis

**Figure 5:**
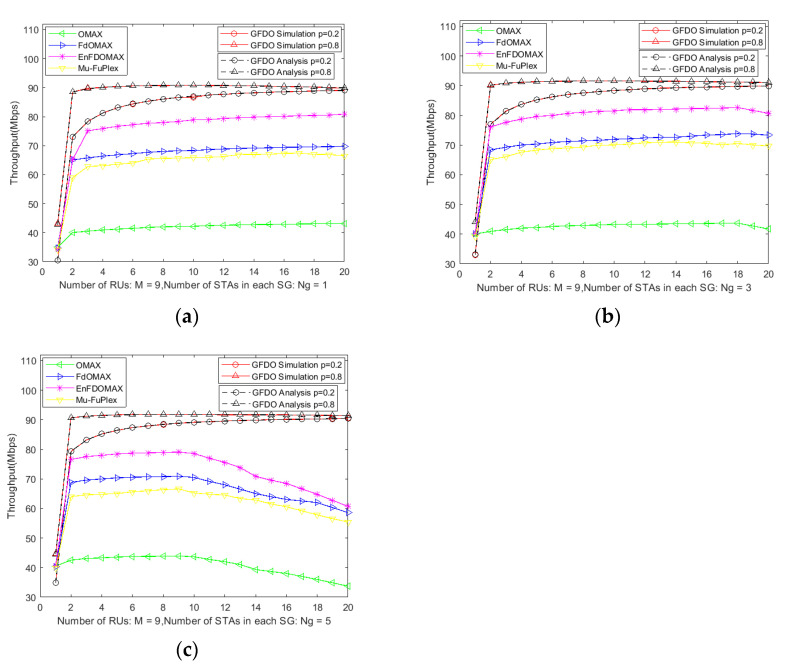
Analysis of system throughput under different *P*-probability. (**a**) Throughput vs. Number of STAs in each SG is 1; (**b**) Throughput vs. Number of STAs in each SG is 3; (**c**) Throughput vs. Number of STAs in each SG is 5.

System throughput is an important performance metric to verify MAC protocol design. The purpose of the simulation is to verify the network throughput trend of GFDO protocol with the increase in node scale, and to compare with the theoretical analysis. From Figure 5, it can be seen that the simulation results are basically consistent with the theoretical analysis, further verifying the correctness of GFDO protocol, where the number of STAs in the SG is 1, 3, and 5 in Figure 5a–c, respectively.

As shown in Figure 5a–c, when the number of GHs is 1, all of the comparison protocols do not form full duplex link transmission, and the access efficiency of OMAX, EnFD-OMAX, FD-OMAX and Mu-FuPlex protocols is higher than the low *P*-probability access mode of GFDO protocol, and their throughputs are higher than GFDO protocol. However, with the increase in GHs and GMs, the randomness of competing nodes in EnFD-OMAX, FD-OMAX and Mu-FuPlex protocols reduces the probability of successfully forming full duplex link transmission, thus affecting the system throughput as shown in Figure 5b,c. The GFDO protocol divides the nodes into several SGs, and GMs in all of SGs compete independently and synchronously, which reduces the probability of competing channels at the same time. In addition, the GFDO protocol uses the information of GH interference to establish multi-user full duplex transmission in each SG, which improves the probability of successful full dual link transmission, thus greatly improving the system throughput. When the network scale reaches a certain number, as shown in Figure 5a, the throughput of OMAX, EnFD-OMAX, FD-OMAX, Mu-FuPlex protocols reduce sharply due to the aggravation of competition conflicts, while the GFDO protocol uses a two-level BSR information collection mechanism to divide the large-scale network into several small-scale networks, reducing competition conflicts, and the simulation results further verify that the GFDO protocol is applicable to the next-generation high-density and EHT WLAN scenarios.

#### 6.2.3. Area Throughput Analysis

**Figure 6:**
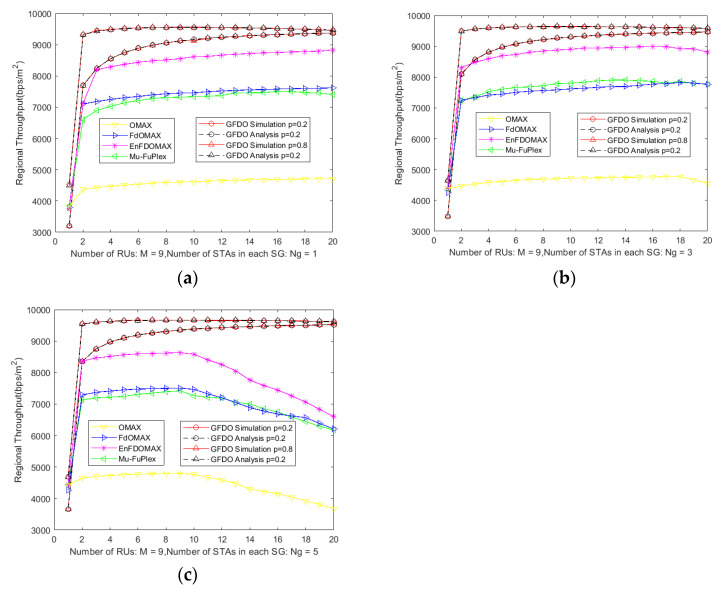
Analysis of area throughput under the number of different nodes. (**a**) Area throughput vs. Number of STAs in each SG is 1; (**b**) Area throughput vs. Number of STAs in each SG is 3; (**c**) Area throughput vs. Number of STAs in each SG is 5.

It can be seen from Figure 6 that the area throughput simulation results of GFDO protocol is consistent with the theoretical analysis, further verifying the correctness of GFDO protocol, where the number of STAs in the SG is 1, 3, and 5 in Figure 6a–c, respectively. When the number of nodes is small, the area throughput of all protocols is on the rise. From the simulation curve, it can be seen that the area throughput of GFDO protocol is improved due to other protocols, on the one hand, the access efficiency of GFDO protocol is improved, and the throughput is improved, which leads to the area throughput improvement. On the other hand, when forming a group full duplex link transmission, because the nodes are grouped according to NCSC, the users of parallel access are relatively concentrated, which reduces the interference diffusion problem, thus improving the area throughput.

As shown in Figure 6c, with the increase in node scale, the system throughput of other protocols decreases sharply due to the aggravation of competition conflicts, which leads to the decrease in area throughput. In the next-generation high-density deployment WLAN scenario, when the OMAX, FD-OMAX, EnFD-OMAX, Mu-FuPlex protocols perform multi-user parallel transmission, the location is relatively scattered, increasing the interference to the surrounding nodes, resulting in a sharp decline in area throughput.

## 7. Conclusions

Aiming at the next-generation high-density deployed WLAN scenario, a spatial Group-based multi-user Full Duplex OFDMA (GFDO) MAC protocol was presented, to jointly solve both the low access efficiency problem and the interference diffusion problem. This protocol divides STAs into several spatial groups without interference, and obtains two advantages. Firstly, the high competition collision probability in high-density deployment scenario is reduced. Secondly, the formation probability of the full duplex transmission pair between the non-interference spatial groups is greatly improved. The simulation results show that the MAC efficiency of the proposed GFDO protocol is 16.8% higher than that of the EnFD-OMAX protocol. In the future, we will focus on the enhancement of GFDO protocol in the optimization algorithm of *P*-probability access and experiment to verify the validity of GFDO protocol.

## Figures and Tables

**Table 1:** Comparison of MAC protocol for Co-time Co-frequency Full Duplex (CCFD) systems.

Reference	Type	Topology	Contention Based	Performance Metric	Key Features	Evaluation
FDC [19]	Symmetric	Centralized	Rotation	Throughput	Scheduling BSR collection on subchannels	Simulator
FD-CSMA/CD [20]	Symmetric	Distributed	Random access	Throughput	Random contention access on subchannel VMAC-hdr	Simulator
FuPlex [21]	Asymmetric	Distributed	RTS/CTS handshaking	Throughput Delay	Next-generation WLAN full duplex MAC framework	NS2 network simulator
Mu-FuPlex [22]	Asymmetric	Centralized	UORA	Throughput MAC Efficiency	Multi-users OFDMA	NS2 network simulator
FD-OMAX [24]	Asymmetric	Distributed	Random access	Throughput FD Link Efficiency	Multi-users OFDMA Inter-node interference collection	NS2 network simulator
EnFD-OMAX [25]	Asymmetric	Distributed	Random access	Throughput FD Link Efficiency MAC Efficiency	Multi-users OFDMA Inter-node interference collection full-duplex link pair matching algorithm	NS2 network simulator
PCMu-FuPlex [23]	Asymmetric	Centralized	UORA	Throughput	Multi-users OFDMA Power Control	NS2 network simulator
GFDO	Asymmetric	Centralized	*P*-probability	Throughput Access Efficiency Area Throughput	Multi-users OFDMA Spatial Group parallel BSR collection, Inter-node interference collection	NS2 network simulator

**Table 2:** Network parameter configuration.

Parameters	Value
Preamble Length	20 μs
PHY Rate	58.5 Mbps
RU Number	9
$SINR_{Throld}$	6 dB
DIFS	34 μs
SIFS	16 μs
Slot	9 μs
TXOP	0.003 s
Bandwidth	80 MHz
